# Peripheral eosinophilia and eosinophilic colitis during long-term azole therapy for pulmonary aspergillosis

**DOI:** 10.1099/jmmcr.0.004135

**Published:** 2014-12-01

**Authors:** Barbara Grzegorczyk, Yoshihiko Murata

**Affiliations:** ^1^​ Saba University School of Medicine, Dutch Caribbean; ^2^​ Division of Infectious Diseases, Department of Medicine, University of Rochester School of Medicine and Dentistry, New York, USA; ^3^​ Infectious Disease Unit, Rochester General Hospital Rochester, New York, USA

**Keywords:** eosinophilic colitis, peripheral eosinophilia, posaconazole, pulmonary aspergillosis, voriconazole

## Abstract

**Introduction:**

Voriconazole and posaconazole are often used to treat invasive *Aspergillus* infections. We describe a patient with chronic pulmonary aspergillosis (CPA) who experienced peripheral eosinophilia and eosinophilic colitis while on voriconazole, and also experienced gastrointestinal symptoms and recurrent eosinophilia while on subsequent posaconazole therapy.

**Case presentation:**

A 75-year-old female with recurrent pulmonary mucus plugs due to CPA was treated with long-term oral voriconazole. The patient had no clinical evidence of CPA exacerbations while on such antifungal treatment but developed peripheral eosinophilia, diarrhoea and eosinophilic colitis after >5 years of voriconazole therapy that resolved after cessation of azole therapy. Due to a CPA exacerbation after stopping voriconazole, the patient was started on posaconazole as an alternative CPA therapy. However, after 15 months, the patient developed a recurrence of peripheral eosinophilia and diarrhoea while on posaconazole.

**Conclusion:**

Long-term use of voriconazole and posaconazole can be used successfully to reduce the incidence of CPA exacerbations. However, such antifungal therapy may also lead to peripheral eosinophilia, diarrhoea and eosinophilic colitis.

## Introduction

Voriconazole and posaconazole are often used for off-label treatment of *Aspergillus* infections such as chronic pulmonary aspergillosis (CPA) (Nagappan & Deresinski, 2007; Saravolatz *et al*., 2003). These azoles are available in oral formulations that facilitate long-term administration and have been shown to be clinically effective in patients with CPA (Sambatakou *et al*., 2006; Kousha *et al*., 2011; Al–shair *et al*., 2013). We report a case of a patient with CPA who developed peripheral eosinophilia and eosinophilic colitis after 5 years of oral voriconazole therapy. Her symptoms and eosinophilia resolved after discontinuing voriconazole and a short course of systemic steroids. Due to a recurrent flare of CPA, the patient was then started on oral posaconazole and 15 months thereafter developed recurrent peripheral eosinophilia and diarrhoea that resolved with discontinuation of the azole.

## Case report

In March 2007, a 75-year-old female with history of hypertension and hypothyroidism presented with a 5-week history of non-productive cough and dyspnoea and was found to have a left lower lobe collapse on a chest X-ray. Her past medical history was significant for a 19-year history of recurrent pulmonary lobar collapse from mucus plugs with cultures positive for *Aspergillus* and thus bore a diagnosis of CPA. She had no documented history of wheezing, peripheral eosinophilia or obvious clinical response to oral steroids, and thus was deemed unlikely to have allergic bronchopulmonary aspergillosis (Kousha *et al*., 2011). Although she had previously been treated intermittently with itraconazole, the patient had not been on any antifungal therapy for 6 weeks prior to presentation due to difficulty in maintaining therapeutic levels of itraconazole to treat pulmonary aspergillosis (Lestner & Hope, 2013). The patient’s radiographic abnormalities and clinical symptoms resolved after a mucus plug was removed by bronchoscopy, and subsequent cultures from mucus material grew *Aspergillus fumigatus*. To reduce the risk of recurrent bronchial collapse due to CPA, the patient was started on oral voriconazole 200 mg twice daily. For the next 5 years, her respiratory status was stable while on voriconazole and without any subjective complaints, respiratory symptoms or laboratory abnormalities.

**Fig. 1.**
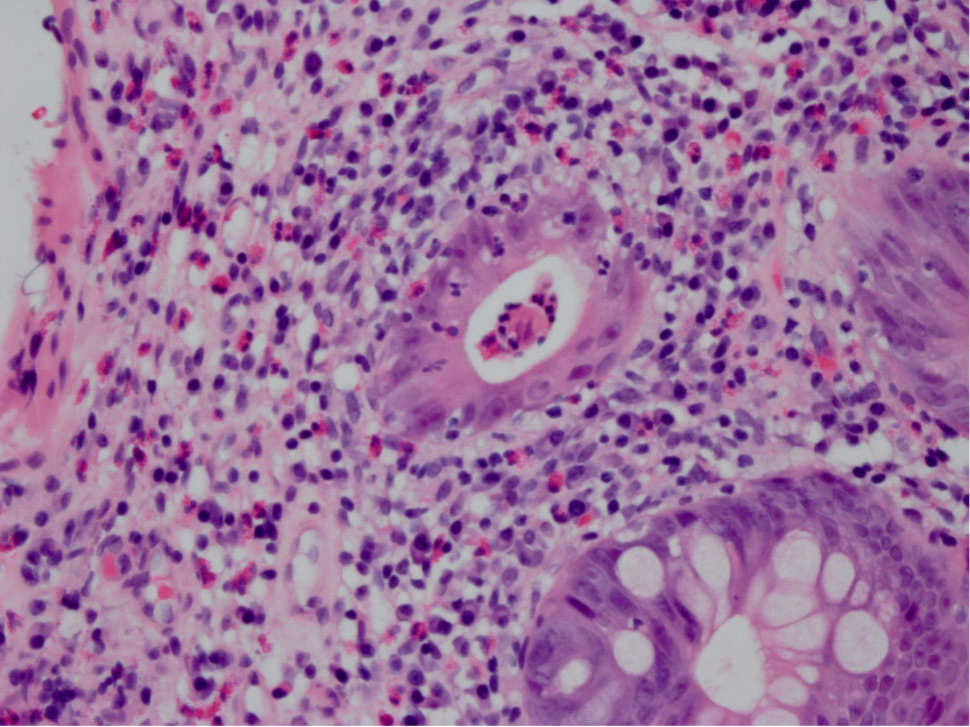
Haematoxylin and eosin stain of a colon biopsy (June 2012) demonstrating colonic inflammation and eosinophilic infiltration.

However, in June 2012, the patient was hospitalized with a 3-month history of progressive abdominal pain, nausea and diarrhoea without any associated respiratory symptoms. She was afebrile and her white blood cell (WBC) count was 9.4×10^3^ μl^−1^ with 15 % eosinophils. Concurrent medications included hydrochlorothiazide and levothyroxine that had been well tolerated for at least several years prior to the start of voriconazole therapy. A contrast-enhanced abdominal computed tomography scan revealed mild thickening and adjacent inflammatory stranding in the ascending colon. Empirical ciprofloxacin and metronidazole were started for presumed intra-abdominal infection. On the day after presentation, her WBC was 11.5×10^3^ μl^−1^ with 49 % eosinophils. Her stool workup was negative for ova and parasites, *Clostridium difficile*, *Giardia*, *Cryptosporidium*, *Salmonella* and *Shigella*. Six days after presentation, a colonoscopy with biopsy was performed and the patient was found to have active chronic colitis with eosinophilic infiltrates and with no evidence of pseudomembranous colitis (Fig. 1). Voriconazole, ciprofloxacin and metronidazole were discontinued and oral prednisone 60 mg once daily was started with rapid improvement in her gastrointestinal symptoms. She was diagnosed with eosinophilia/eosinophilic colitis presumably due to voriconazole. We believe that the probabilistic causality between the observed eosinophilia/eosinophilic colitis and voriconazole is deemed probable based on a score of 6 using the Naranjo algorithm (Naranjo *et al*., 1981). At her follow-up visit 18 days after hospitalization, her symptoms had completely resolved with a WBC count of 8.7×10^3^ μl^−1^ with 0 % eosinophils.

The patient was asymptomatic without antifungal therapy until November 2012, when she presented with several weeks’ history of shortness of breath. A chest X-ray revealed right lower and middle lobe collapse due to mucus plugs that were removed during bronchoscopy and subsequently grew *Aspergillus terreus*. As her recurrent respiratory symptoms strongly suggested a recrudescence of CPA while off antifungal therapy, the patient was started on oral posaconazole 200 mg twice daily instead of the labelled dosing (in the USA) of 200 mg three times daily (Nagappan & Deresinski, 2007). The dose reduction of posaconazole was discussed with the hospital pharmacy and agreed with the patient, and was primarily due to her age (80 years old at the start of posaconazole therapy) and weight (∼45 kg), and after careful discussion with the patient regarding the potential benefits versus the risk of cross-reactive adverse events, i.e. eosinophilia, while on posaconazole. She was asymptomatic for 15 months but then presented with a 2-week history of progressive diarrhoea and concurrent peripheral eosinophilia of 15 %. A presumptive diagnosis of azole-associated eosinophilia and diarrhoea was made and her symptoms resolved after stopping posaconazole. At the time of writing, the patient remains asymptomatic and off antifungal therapy.

## Discussion

We have described a patient with CPA who developed peripheral eosinophilia and eosinophilic colitis that developed after 5 years of treatment with oral voriconazole and diarrhoea as well as peripheral eosinophilia and diarrhoea after 15 months of treatment with oral posaconazole.

We are aware of one previously published case of voriconazole-induced peripheral eosinophilia with fever, leukocytosis of 78×10^3^ mm^−3^ and eosinophilia of 10.6 % on day 11 of intravenous voriconazole for the treatment of *Aspergillus* lung infection in a patient after bone-marrow transplant (Vishnubhotla *et al*., 2004). Within 2 days of stopping the azole, the patient’s fever resolved and the WBC decreased to 10.5×10^3^ mm^−3^ with 5.7 % eosinophils. In contrast to this previous case, our patient developed eosinophilia only after prolonged voriconazole therapy of over 5 years’ duration, which was tolerated with no clinically significant adverse events until the onset of eosinophilia with gastrointestinal symptoms. Due to the resolution of eosinophilia and diarrhoea upon cessation of voriconazole and administration of steroids, and with no other obvious cause of peripheral eosinophilia, we believe that our patient’s signs, symptoms and laboratory abnormalities were the result of voriconazole treatment.

A distinguishing feature of our case was the histopathological analysis of the patient’s colon biopsy, which revealed chronic and active eosinophilic colitis with increased numbers of eosinophils (Fig. 1). The patient was not known to have a prior history of primary or secondary eosinophilic colitis and her concomitant medications had not changed in several years. We speculate that the marked peripheral eosinophilia caused by an allergic response to voriconazole also led to a concomitant colonic tissue infiltration and the symptoms of abdominal pain and diarrhoea. To our knowledge, azole-induced eosinophilic colitis has not been reported previously.

The patient also developed peripheral eosinophilia and diarrhoea after 15 months of treatment with another triazole, posaconazole. It is possible that peripheral eosinophilia and diarrhoea may represent adverse effect profiles that are shared by voriconazole and posaconazole due to similarities in their chemical structures (Nagappan & Deresinski, 2007; Saravolatz *et al*., 2003). To the best of our knowledge, this is the first description of posaconazole-induced eosinophilia and diarrhoea.

In conclusion, clinicians should consider monitoring eosinophil levels in patients receiving long-term azole antifungal agents, and eosinophilic colitis should be part of the differential diagnosis in such patients who develop gastrointestinal symptoms.

## Abbreviations

CPA: chronic pulmonary aspergillosis
WBC: white blood cell.
